# The role of a pseudocapsula in thymic epithelial tumors: outcome and correlation with established prognostic parameters. *Results of a 20-year single centre retrospective analysis*

**DOI:** 10.1186/1749-8090-4-33

**Published:** 2009-07-15

**Authors:** Sebastian Dango, Bernward Passlick, Ulf Thiemann, Gian Kayser, Christian Stremmel

**Affiliations:** 1 Clinic for Thoracic Surgery, Hugstetter Str. 55, University Hospital Freiburg, Albert-Ludwig-University, 79106 Freiburg, Germany; 2 Pathological Institut of University Hospital Freiburg, Breisacherstr. 115, Albert-Ludwig University, 79106 Freiburg, Germany

## Abstract

**Background:**

Treatment of thymoma is often based on observation of only a few patients. Surgical resection is considered to be the most important step. Role of a pseudocapsula for surgery, its clinical significance and outcome compared with established prognostic parameters is discussed which has not been reported so far.

**Methods:**

84 patients with thymoma underwent resection and analysis was carried out for clinical features, prognostic factors and long-term survival.

**Results:**

Fifteen patients were classified in WHO subgroup A, 21 in AB, 29 in B and 19 patients in C. Forty two patients were classified in Masaoka stage I, 19 stage II, 9 stage III and 14 stage IV. Encapsulated thymoma was seen in 40, incomplete or missing capsula in 44 patients. In 71 complete resections, local recurrence was 5%. 5-year survival was 88.1%. Thymomas with pseudocapsula showed a significant better survival (94.9% vs. 61.1%, respectively) (p = 0.001) and was correlated with the absence of nodal or distant metastasis (p = 0.04 and 0.001, respectively). Presence of pseudocapsula as well as the Masaoka and WHO classification, and R-status were of prognostic significance. R-status and Masaoka stage appeared to be of independent prognostic significance in multivariate analysis.

**Conclusion:**

Intraoperative presence of an encapsulated tumor is a good technical marker for the surgeon to evaluate resectability and estimate prognosis. Although the presence of a capsula is of strong significance in the univariate analysis, it failed in the multivariate analysis due to its correlation with clinical Masaoka stage. Masaoka stage has a stronger relevance than WHO classification to determinate long-term outcome.

## Introduction

Although thymoma is with 50% the most common tumor of anterior mediastinum clinical management has been based largely on observations in a few single-centre experiences. The largest studies were performed in Japan. Thymomas are compulsory malignant tumors with an incidence of 0.2 – 1.5% worldwide [1]. A larger body of literature has been presented in the last decades providing further insight into tumor biology and clinical behaviour of thymomas [2]. Only a few reports include a significant number of patients with thymomas that have followed up for a sufficient five year period. Regarding the clinical approach different concepts are emerging as a result of a more evidence-based clinical management. Surgery is the gold standard of treatment. Herein, we present a 20 year experience in clinical treatment of thymomas as a result of a retrospective single centre analysis done within a European university setting. The role of a pseudocapsula and its clinical significance and outcome compared with established prognostic parameters is discussed which has not been reported so far.

## Materials and methods

### Patients

Patient data were collected using a questionnaire that was developed for this study and approved by the local ethics committee. A retrospective review of surgical records at the Department of Thoracic Surgery, University of Freiburg identified a total number of 84 patients who had received surgical treatment for thymoma between 1984 and 2004. Only histologically confirmed cases were included. A performed neoadjuvant or adjuvant treatment was documented. Follow-up data were obtained through the department's archives or, after informed consent, the patients themselves or, if deceased, their relatives or family physicians.

Overall 46 patients were male, 38 were female. The age of the patients ranged from 14 to 82 years (median 58 years). Also patients with a neoadjuvant or adjuvant treatment were included in the study. Two patients were excluded for further analysis because of incomplete follow-up; overall 82 patients were introduced to survival analysis.

### Clinical Pathology and Immunhistochemical Staging

Hematoxylin-eosin-stained sections of all patients were available for the re-evaluation of the histologic diagnosis according to the commonly known WHO schema of 1999 [2-4]. Additionally special immunhistochemical staining was performed for Zytokeratin, Neuron-specific enolase (NSE), cluster of differentiation (CD) 1a, 3, 5, 20 and 117 (c-kit). The histological evaluation was carried out by two independent senior pathologist at the Department of Pathology, University of Freiburg, without any information about the patient's clinical features.

The classification proposed by Masaoka was adopted to determine the tumor stage as described elsewhere [5,6]. This staging system is based on the extent of macroscopic or microscopic invasion in mediastinal structures. The clinical stage was thus determined by critical review of surgical records and pathology reports. Further more, R-, N- and M-Status was classified in addition to a local infiltration of vessels and pericardium and the presence of a pseudocapsula was taken into analysis. Possible other clinical relevant features such as myasthenia gravis were recorded as well as surgical complications.

### Statistical Analysis

For all statistical analysis SPSS 14.0 software (SPSS Inc., Chicago, IL, USA) was used. *p *< 0.05 was assumed significant unless otherwise stated. The above named perimeters were taken into statistical investigation and possible correlation was analysed using Pearson's Chi^2^-test. Overall survival time was calculated from the date of surgery to death or last follow-up. Observations of living patients were arrested on database and continually actualised. Overall subgroup for each spectrum was determinate and a survival analysis was performed. Deaths related to the tumor were considered events; all deaths not related were considered as censored observations. Survival rates were calculated with the Kaplan-Meier method, statistical difference in survival was determined by using the log-rank test and in case of significance a univariate analysis and Cox-regression analysis was carried out.

## Results

### Pathological and Clinical Findings

According to the WHO classification 15 tumors were classified as type A, 21 tumors were type AB, 13 patients with type B1, 13 with type B2, 3 type B3 tumors and 19 (20%) patients with type C tumors. Out of these 19 types C tumors 5 (25%) were histologically diagnosed as thymic carcinoid. Clinical staging according to Masaoka showed that 42 patients were classified in stage I, 19 patients in stage II, 9 in stage III and 8 and 6 were referred to IVa or IVb, respectively (Table 1). 85% of the patients staged in WHO A-B2 were classified in Masaoka stage I and II.

**Table 1:** Clinical features of different thymoma subtypes classified according WHO^a^

**WHO**	**n**	**Age (yr)**	**MG**^**b**^**(%)**	**Weight (g)**	**Masaoka** **I**	**Masaoka** **II**	**Masaoka** **III**	**Masaoka** **IV**
A	15	64.0 ± 9.8	26.6	164.0 ± 293.0	10	4	1	0
AB	21	58.5 ± 10.6	14.2	114.0 ± 131.4	15	2	4	0
B1	13	58.6 ± 6.3	23.0	103.0 ± 99.5	9	2	1	1
B2	13	51.8 ± 20.3	53.0	125.0 ± 108.6	5	6	0	2
B3	3	67.8 ± 23.4	66.6	166.0 ± 83.5	1	2	0	0
C	19	54.1 ± 13.4	0	139.0 ± 403.4	2	3	3	11
Total	84	-	22.6	-	42	19	9	14

^a^Data are presented as mean ± SD unless otherwise indicated.

^b^Myasthenia gravis

Nineteen patients presented with myasthenia gravis. Other clinical features were as follows: chest discomfort (10 patients), thoracic pain (7), weight loss (3), or other symptoms (vena cava superior syndrome, bleeding). Thirty two patients did not present any clinical symptoms and in 5 cases eventually presences of clinical symptoms were unclear.

Complete resection (R0) was achieved in 71 patients (84%), R1- and R2-resections were carried out in 5 (6%) and 8 (10%) patients, respectively (Table 2).

**Table 2:** Clinical features of different thymoma classified according Masaoka Stage^a^

**Masaoka**	**n**	**R0-status**	**N1**	**M1**	**Recurrence**	**5-year survival (%)**
I	42	42 (100%)	0 (0%)	0 (0%)	0 (0%)	97.6
II	19	18 (94.7%)	0 (0%)	0 (0%)	1 (5.3%)	94.7
III	9	6 (66.7%)	0 (0%)	0 (0%)	0 (0%)	66.7
IV	14	5 (35.7%)	6 (42.8%)	8 (57.1)	1 (7%)	64.3

^a^Data are presented as mean ± SD unless otherwise indicated

Local recurrence depended on the stage and was noted in 2 patients (1.7%). 1 patient in Masaoka stage II appeared to have a local recurrence after 79 month, another patient in Masaoka stage IVa after 11 month. Both, R-status and local recurrence were dependent on the Masaoka stage (p < 0.001).

An involvement of adjacent anatomical structures was found overall in 53 patients including the pleura, pericard, vessels or neighboured lung tissue. All patients were staged Masaoka IV, only one case with involvement of the vena anonyma was staged Masaoka III. Two patients presented with an infiltrating tumor of pericardium and nervus phrenicus. Infiltration of pericardium was correlated with the WHO classification (p < 0.001).

Encapsulated tumor with presence of a complete or incomplete pseudocapsula was identified in 66 patients (78.6%). In detail, with 40 patients the majority of thymomas were encapsulated totally, 26 were incomplete structured and in 18 cases presence of a capsula was missing. A correlation was seen between the presence of a pseudocapsula and Masaoka as well as WHO classification (p = 0.001) (Table 3). Presence of a pseudocapsula had influence on N-status, M-status, infiltration of pericardium or surrounded vessels and local recurrence (Table 3). In 79 patients there was no lymph node involvement and in 5 cases a local lymph node metastasis was found (N1). None presented with an ipsilateral (N2) or clinical seen contralateral lymph node metastasis (N3). Distant metastases (M1) were collected in 8 patients with thymic tumors. Eight metastases were found; four patients appeared to have a systemic disease with multiple metastases. Single metastasis was situated either in the pleura, liver or lung. Pleural metastasis was seen in 1 case, further more 2 metastases were found in the lung and 1 metastasis in the liver. Recurrence was only seen in one case each in patients with an incomplete encapsulated thymoma or a missing capsula.

**Table 3:** Patient characteristics and tumor parameters according to the presence of a pseudocapsula^a^

**Characteristics**	**n**	**Pseudocapsula** **complete**	**Pseudocapsula** **incomplete**	**Pseudocapsula** **no capsula**	***p-value***^***b***^
Masaoka I	42	38	4	0	**0.001**
II	19	1	16	2	
III	9	1	3	5	
IV	14	0	3	11	
N-status N0	79	40	24	15	**0.042**
N1	5	0	2	3	
M-status M0	76	40	25	11	**0.001**
M1	8		1	7	
Vessel infiltration	13	0	3	9	**0.001**
Pericard infiltration	14	1	1	12	**0.001**
Recurrence	2	0	1	1	**0.047**
Age (yr)^c^	84	40	26	18	0.44
Sex Male	45	14	17	14	**0.004**
Female	39	26	9	4	
MG^d^	19	10	6	3	0.86

^a^Data are presented as mean ± SD unless otherwise indicated

^b^Two-sided *p *values were calculated by Pearson's Chi-Square test to determine the significance of correlation of clinicopathological parameters and presence of a pseudocapsula of the tumor.

Boldprinting indicates statistical significance

^c^Range from 14 to 82 years

^d^Myasthenia gravis

### Neo-Adjuvant and Adjuvant Therapy

As far as a neoadjuvant or adjuvant therapy is concerned two major aspects must be figured out. First, clear indications are essential, second, a potential benefit for subgroups must be analysed. Therefore, we performed subgroup analyses and evaluated clinical findings as present Masaoka stage compared to given treatment to define possible benefit for survival.

8 patients were included into a neoadjuvant treatment regime inclusive chemotherapy and/or radiation therapy, 33 patients were taken into an adjuvant therapy regime. In detail, 3 patients were either treated alone with neoadjuvant chemotherapy or radiation alone, one patient received combined treatment, and in another patient an immunotherapy was performed. Adjuvant radiation was executed in 26 cases, chemotherapy alone was given in 5 cases, and a combination of both was performed in 2 patients. Interestingly, 2 patients in stage IV were resected R0 after neoadjuvant chemotherapy which was carried on after surgery. No immunotherapy was carried out as adjuvant treatment regime. Five patients were taken to adjuvant systemic therapy alone and 2 patients received combined radiotherapy in stage IV. No chemotherapy was performed in Masaoka stage I-III. There was no local recurrence in patients staged Masaoka I or III in our study group.

Patients with complete encapsulated thymoma did not receive neoadjuvant treatment, only one patient needed radiation after surgery. 44 patients with incomplete or missing capsula were taken into neoadjuvant or adjuvant treatment. In detail, 7 patients without a capsula received neoadjuvant including immunotherapy in one case. 25 patients (15 with incomplete capsula and 10 with no capsula) were taken into adjuvant radiation, 2 patients with no capsula into ongoing chemotherapy after surgery.

### Survival Analysis and Prognostic Factors

**Figure 1:**
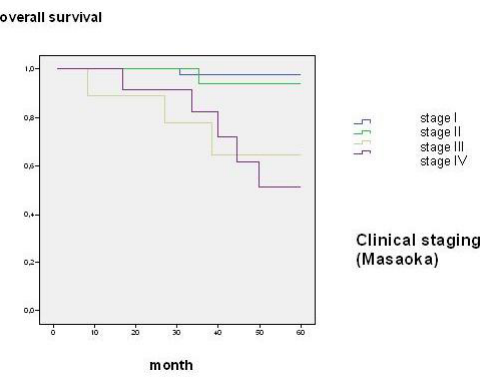
**Overall survival according to clinical Masaoka stage**.

**Figure 2:**
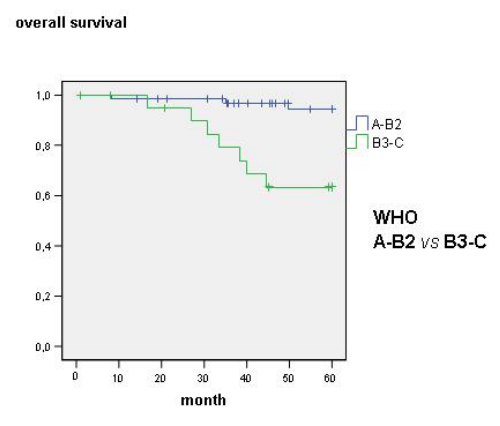
**Overall survival according WHO classification A-B2 and B3-C**.

**Figure 3:**
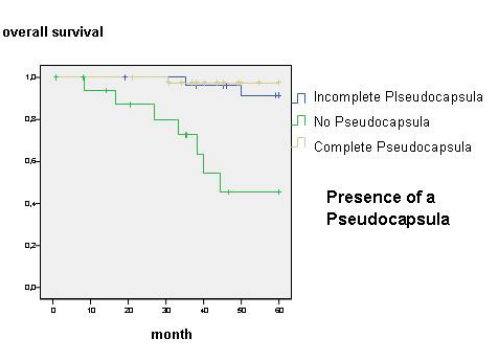
**Overall survival according presence of complete/incomplete capsula**.

**Figure 4:**
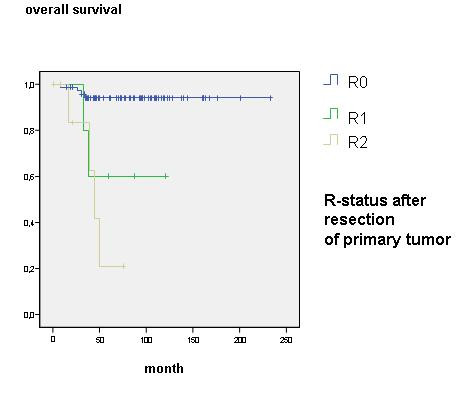
**Overall survival according resection status (R-status)**.

For incomplete follow-up or cancer-unrelated death two out of 84 patients were excluded from survival analysis and 82 patients were determined for further investigation. Overall survival was 88.1% after 45 month. Age, sex, clinical staging through Masaoka, WHO classification, R-status, encapsulated or non-encapsulated thymoma as well as the N- and M-status and local recurrence was statistically reviewed. Local recurrence for patients with a performed R0-resection was found in 2 cases (2.7%) within follow-up of 45 month and had no influence on overall survival. In Masaoka stage I an overall survival rate of 59.2 month, in stage II of 58.5 month, in stage III of 47.7 month and in Masaoka stage IV survival rate of 49.8 month were found (Fig. 1). The 5-year survival rate decreased from over 94% in Masaoka stage I and II to less than 67% in Masaoka stage III and IV (Table 2) WHO classification reflects decreased overall survival in further ranked WHO classes (p = 0.003, figure not shown). Subgroup analysis showed an unfavourable outcome with significant difference in class A-B2 and B3-C (p < 0.002, 58 *vs*. 50 month) (Fig. 2). Patients with complete encapsulated tumors lived for 59 month and with incomplete capsula for 58 month, difference was statistically not significant. But patients with a missing pseudocapsula showed an unfavourable outcome compared to an encapsulated tumor with an overall survival of 44 month (p < 0.001) (Fig. 3). An achieved R0-state improves surgical outcome (Fig. 4) with a significant survival advantage for the patient (p < 0.001, std. deviation 4.92, confidence interval 95%: 34.8–54.1). A performed analysis to evaluate a difference between R1- compared to a R2-resection of 13 patients failed showing any relevant difference in patients' outcome (p = 0.39). Automatically, no influence on survival was seen for patients with performed biopsy. Patients with clinical finding of Myasthenia gravis did also not have a survival advantage (p = 0.46). Histological appearance according to Bernatz and Marino or Müller-Hermelink did not appear to have any clinical or prognostic relevance in the performed statistical investigation and was not taken into further analysis (p = 0.25). Also the immunohistochemestry (NSE, Cytokeratin, CD1a, 3, 5, 20 and 117) did not show any prognostic relevance.

Subgroup analysis was carried out to determine an advantage for patients with neoadjuvant or adjuvant treatment. Subgroup analysis showed only significant favourable clinical outcome by adjuvant therapy for patients with thymic tumors in Masaoka Stage III (p = 0.014).

Factors of prognostic impact were evaluated in the population. Present symptoms, patient's age, as well as resected tumor mass, possible lymph node involvement and distant metastasis and infiltration of adjacent structures did not have any prognostic influence. Regression analysis demarked a significant correlation only for vessel infiltration and prognosis (p = 0.027). Strong prognostic impact was found for Masaoka staging system (p < 0.001). WHO classification stresses poor prognosis of related patients (p = 0.002). Surgical outcome measured by resection state appeared as the strongest significant prognostic parameter in the population (p < 0.001). Multivariate analysis was performed to figure out possible correlations with WHO classification and Masaoka Staging system as well as presence of a pseudocapsula. Joint-effects were analysed by Cox-Regression and independence was found for resection state and Masaoka (Table 4). An increased risk for increased cancer-related death of almost four times was figured out for incomplete compared to complete resection. A relative risk for decreased survival of over two was found for increased Masaoka stage of patients with thymoma. WHO reflects biological behaviour as well as tumors' aggressiveness and went confirm with Masaoka staging and clinical outcome not being of prognostic independence.

**Table 4:** Univariate and multivariate analysis of cancer-related survival in the total population^a^

**Risk factor**	**Univariate analysis**	**Multivariate analysis**^**c**^		
	***p *value**^**b**^	**Relative risk**	**95% Confidence interval**	***p *value**

R-status	0.0001	3.9	2.0 – 7.6	**0.001**
Masaoka staging^d^	0.0001	2.0	1.0 – 4.1	**0.020**
Presence of a pseudocapsula	0.0002	-	-	0.179
WHO^d ^classification^e^	0.0001	-	-	0.181
Sex	0.562	-	-	0.849
Age	0.360	-	-	0.206

^a^Cancer-unrelated death or incomplete follow-up resulted in exclusion of 2 patients leaving 82 patients available for the analysis of joint effects of prognostic parameter.

^b^*p *values of univariate analysis were determined by Log rank test.

^c^Stepwise multivariate analysis was performed using the Cox prpotional-hazard model.

^d^WHO classification was grouped into two major classes: A-B2 and B3-C.

^e^Masaoka and WHO did show joint effects with pseudocapsula and were therefore not taken to further analysis.

An encapsulated tumor presented a better clinical outcome than loss of a pseudocapsula (p < 0.001). Thymoma with complete or incomplete capsula showed a statistically significant better survival than without pseudocaosula (94.9% vs. 61.1%; p = 0.001) which reflects the Masaoka staging system. Survival analysis showed a better outcome after surgery in patients with encapsulated tumor which results in less lymph node metastases, less distant metastases and less infiltration of surrounded vessels and pericardium. Patients in Masaoka stage IV had more often a non-encapsulated tumor with increased cancer related death (Table 3). Interestingly, complete or incomplete encapsulated thymomas required less often a neoadjuvant or adjuvant treatment with better prognosis (p < 0.002). This correlation was not independent in the performed multivariate analysis.

Summarized, Masaoka stage is of stronger significance in patients with a thymoma compared to WHO classification. Surgery with complete resection is a very favourable cancer-related prognostic factor in patients with thymoma.

## Discussion

Thymoma is a rare tumor entity and clinical management is very often based on observations of only a few patients in a single center. Surgery is considered the mainstay of therapy and recurrence is described as a typical nature of this tumor [1,3,7-11]. Presence of a pseudocapsula influences therapy regime and clinical outcome. Thymomas vary in its biological behaviour; also biology of this malignancy is still not fully understood. Two major classifications for thymomas are important and help to find the best therapy adapted to the prognosis.

In this series we found an overall survival of 88.1% which is representative to described data in the literature [1-3,5,7-12]. Vessel infiltration as well as Masaoka stage, WHO classification, R-status and an encapsulated tumor were of prognostic significance. Multivariate analysis was carried out to analyze possible joint-effects of prognostic parameter and only R-status and Masaoka stage appeared to be of independent prognostic significance in our series.

Even though being of strong significance in the univariate survival analysis WHO classification was not independent in the Cox-regression analysis. For clinical use and estimation of the patients' prognosis WHO classification is not as useful as the Masaoka classification which was shown before [3]. The reason for these is based in a couple of problems. First, there is a significant interobserver variability in histological typing [2,6]. Second, determination of precise cut-off points between different categories (e.g. B1 to B2, or B2 to B3) may lead to different categorisation, especially in highly biological active tumors such as B3 thymomas [3]. Third, proportion of the invasive tumors is not reflected in the WHO classification, and therefore, prognostic value is not preciously mirrored [4,13].

Kim reported a simplification classifying thymomas into different groups; A-B2 on the one side and B3 and C thymomas on the other side [3]. We used this simplification for statistical analysis and presentation of clinical features (Table 1). It better reflects real survival rates and prognosis, as shown previously [1,3,14]. There is a significant difference between cancer-related survival for patients according to WHO classification A-B2 and B3-C. Prognosis is good in patients with type A to B2 thymomas with no tumor related death. In our cohort survival rate was over 95% for WHO A-B2 and only 68% for B3-C. Several investigators have also reported poor prognosis of type B3 thymoma [1-3,10], whereas no difference is found in others reports in the literature concerning B2 and B3 thymomas [15-17]. In our study group 84% of patients in WHO A to B2 were classified into Masaoka stage I or II. Therefore, the shown joint effect in multivariate analysis due to a large overlap between different subgroups of WHO and early Masaoka stages is easily explained.

Masaoka staging system was the strongest independent factor for survival additional to the R-status in our study group. Large thymomas in advanced Masaoka stages are not very likely to be resected R0 and thus having a decreased outcome [18].

In our study population an encapsulated tumor is associated with a decreased cancer-related survival compared to a thymoma without a capsula. In complete or incomplete encapsulated thymomas survival rate is 97% and 92%, respectively, while survival rate without a capsula is lightly over 60%. Also being of strong significance in the univariate analysis, presence of a pseudocapsula fales as independent prognostic parameter in the multivariate analysis due to its narrow correlation with the clinical Masaoka stage. However, correlation with Masaoka can easily be explained. A pseudocapsula borders the tumor and limits local infiltration and reflects a less aggressive behaviour. Thus, less lymph node or distant metastases and infiltration of adjacent structures like pericardium or vessels are found (Table 3). Therefore, intraoperative presence of an encapsulated tumor is a good technical marker for the surgeon to evaluate resectability and estimate patient's prognosis including recurrence. This reason emphasizes the importance of a capsula for Masaoka staging.

In our population 26 patients were additionally treated with radiation, out of these 2 patients in stage IV were taken into combined radiochemotherapy after surgery. Subgroup analysis showed significant favourable outcome through adjuvant therapy only for patients with thymic tumors staged Masaoka III. Thymomas are radiosensitive, and radiotherapy (RT) is generally accepted for advanced stages after partial resection [19-22]. However, strong evidence for this is still missing and efficacy is hard to define because of small numbers of patients in the presented studies. Therefore, whether adjuvant RT should be given after resection remains controversial, especially all series addressing postoperative RT involve retrospective reviews including many decades rather than an experience with a defined treatment plan and selection criteria [23]. In complete resected thymomas recurrence rate for stage I and II is so low without adjuvant RT, that the use of RT after surgery can not be recommended [7]. For sure, adjuvant radiation is compulsory for incomplete resection also not showing a better survival. But adjuvant radiation was found to result in lower recurrence rate in patients with incomplete resected thymomas in stage IV [23,24].

In our study chemotherapy was given in 5 cases only in stage IV and recurrence rate was 7%. No patients in Masaoka stage I-III received neoadjuvant or adjuvant chemotherapy. We did not find any survival advantage for patients with either neoadjuvant or adjuvant systemic treatment. These findings reflect results presented in the literature. Generally, thymomas in stage III and IV do not appear to have a better outcome for adjuvant chemotherapy compared to surgery alone, but in the biggest presented study so far by Kondo a survival advantage was seen for thymic carcinomas [7].

Multimodality treatment consisting of preoperative chemotherapy, surgery and adjuvant RT was carried out in 2 patients in our study group. There was no survival benefit for this subgroup also the number of patients is extremely small. Interestingly, 2 patients in stage IV were resected R0 after neoadjuvant chemotherapy. Adjuvant treatment was carried on with chemotherapy and therefore leads to a better survival through multimodal therapy. Generally, the position of a multimodality treatment is still discussed controversially because of only small heterogeneous number of patients in the presented literature. A possible resection and survival might be improved in patients with stage III and IV thymomas as reported in the literature [11,25]. Prospective studies showed increased resectability up to 72% with an average 5-year survival rate of 78% [7,11,14]. Therefore, a multimodal treatment regime appears to have a slightly better survival for patients in stage III and IV than for patients with surgery alone, independent, if postoperative RT alone or combined radio chemotherapy is used [7,11,14,25].

Summarized, the shown data confirm the published results on the clinical prognosis of different histological subtypes and the difficulties with the staging systems. Further more, our data point out a difference in survival and prognosis when tumor is complete or incomplete encapsulated stressing the importance of knowledge of presence. According to our opinion, differentiated pathological assessment for pseudocapsula can surely improve clinical evaluation of surgical outcome and is therefore compulsory. The strongest impact on survival is surely a complete resection, which is dependent on Masaoka staging. Prognosis can be evaluated best by carrying out the Masaoka staging. Moreover, WHO classification is not as precise as the Masaoka classification for prediction of prognosis of the patient which is for clinical management still the best. To our opinion pathologically confirmed complete encapsulated tumors do not require any neo- or adjuvant treatment after complete resection. The worse survival results for the higher Masaoka stages support a combined therapy with neoadjuvant chemotherapy and adjuvant radiotherapy which is supported by the literature [14]. More prospective randomized trials are essential to clarify possible beneficial effects for advanced thymomas or thymuscarcinomas.

## Conclusion

Intraoperative presence of an encapsulated tumor is a good technical marker for the surgeon to evaluate respectability and estimate prognosis. Although the presence of a capsula is of strong significance in the univariate analysis, it failed in the multivariate analysis due to its correlation with clinical Masaoka stage. Masaoka stage has a stronger relevance than WHO classification to determinate long-term outcome.

## Competing interests

The authors declare that they have no competing interests.

## Authors' contributions

SD has conceived the study, participated in the design, carried out statistical analysis, mainly composed the manuscript and performed administrational and institutional work. BP is the Head of Department and reviewed the manuscript and gave scientific impact. UT acquired the data retrospectively. GK as pathologist classified thymomas and reviewed histological and immunhistochemical staining of the specimens. CS has participated as senior author in the design of the study and helped to draft the manuscript. All authors read and approved the final manuscript
